# Patent Foramen Ovale Closure for Nonstroke Indications

**DOI:** 10.1016/j.jscai.2023.101135

**Published:** 2023-09-16

**Authors:** Akilesh P. Honasoge, Hussam S. Suradi, Jonathan M. Tobis, Clifford J. Kavinsky

**Affiliations:** a Division of Cardiology, Department of Medicine, Rush University Medical Center, Chicago, Illinois; b David Geffen School of Medicine at UCLA, Los Angeles, California; c Department of Medicine, Beth Israel Deaconess Medical Center, Harvard Medical School, Boston, Massachusetts

**Keywords:** decompression, illness, liver transplantation, migraines with aura, patent foramen ovale closure, platypnea-orthodeoxia syndrome

## Abstract

The patent foramen ovale (PFO) is a flap or tunnel-like communication in the atrial septum present in 20% to 34% of the adult population. In most cases, it is a benign finding and poses no health risk. However, some PFOs may provide a conduit for bloodborne materials, such as thrombi, vasoactive substances, or air to pass into the systemic circulation causing a paradoxical embolus. PFOs have been linked with several clinical disease states including cryptogenic stroke, migraine headache, platypnea-orthodeoxia, and decompression illness. Percutaneous PFO closure provides a practical solution to the problem of PFO in carefully selected populations. Recent randomized control trials have demonstrated that PFO closure in patients with cryptogenic stroke is associated with reduced rates of recurrent stroke compared with medical therapy. This translated into a dramatic increase in the number of PFO closure procedures worldwide, primarily for the indication of cryptogenic stroke, with high procedural success and low complication rates. However, there are no randomized clinical trials available to support PFO closure in other clinical conditions. This article reviews potential indications, existing data, and management approaches for PFO closure in disorders other than cryptogenic stroke.

## Highlights

• Patent foramen ovale closure for migraines does not have evidence to support widespread adoption.
• Patent foramen ovale closure may benefit a subpopulation of refractory migraines with aura.
• Platypnea-orthodeoxia syndrome with right-to-left shunt responds to patent foramen ovale closure.
• Consider conservative dive profiles and cautious patent foramen ovale closure for decompression illness.
• Avoid routine asymptomatic patent foramen ovale closure prior to liver transplantation.

## Central Illustration

**Figure:**
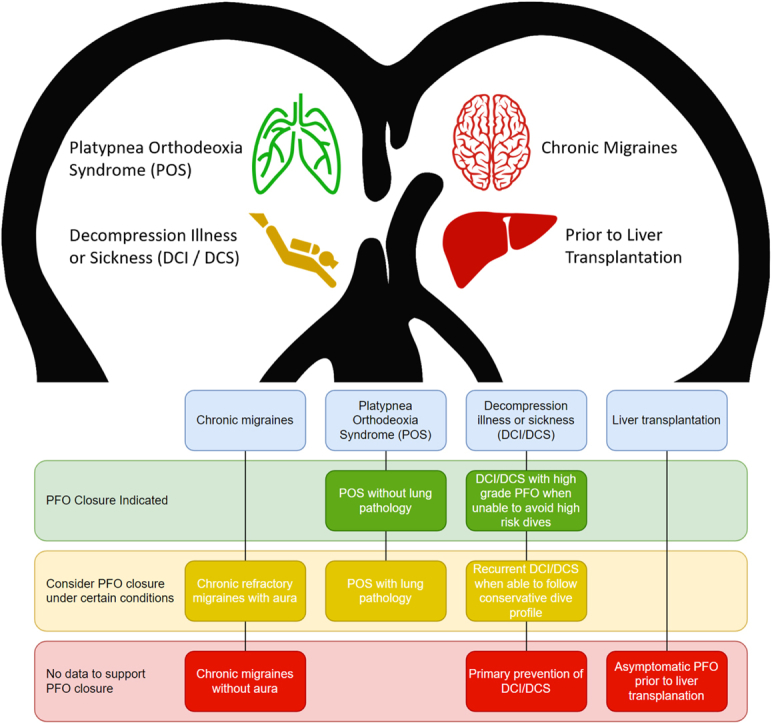

## Introduction

The foramen ovale is an obligatory channel between the atria that allows placental oxygenated blood to reach the arterial circulation during the embryonic period. When there is incomplete postnatal fusion of the septum primum and secundum, a patent foramen ovale (PFO) is formed, which occurs in about a quarter of the adult population.^1^ For the majority of people, a PFO will remain undetected or appear only as an incidental finding during cardiac investigation. However, some PFOs may result in either transient or continuous right-to-left shunt (RLS) that can potentially provide a conduit for bloodborne materials, such as thrombi, vasoactive substances (eg, serotonin, prostaglandins, or nitric oxide), or nitrogen bubbles to pass into the systemic circulation. The morphology of PFO tends to be variable and certain anatomic features such as large defects (>6.5 mm by transesophageal echocardiogram [TEE]), persistent RLS at rest, and presence of atrial septal aneurysm or prominent Eustachian valve have been associated with a greater risk of paradoxical embolism.^2^ The presence of PFO has been associated with a range of clinical disease states such as stroke, migraine headache, platypnea-orthodeoxia, and decompression illness (DCI). With the advent of transcatheter therapies, PFO device closure emerged as a practical solution to the problem of PFO-mediated illness. Recent randomized control trials (RCTs) have demonstrated that PFO closure in patients with cryptogenic stroke is associated with reduced rates of recurrent stroke in carefully selected populations.^3^ This translated into a dramatic increase in the number of PFO closure procedures performed worldwide, primarily for cryptogenic stroke indication, with high procedural success and low complication rates.^1^ However, the outcome of PFO closure in clinical conditions other than stroke, such as migraine, decompression sickness (DCS), and desaturation syndromes, remains largely unknown as many of these clinical scenarios do not lend themselves to RCT design due to the small number of patients and events. Given the lack of RCT data on PFO closure in nonstroke indications, prior guidelines from societies were limited to PFO closure for stroke indication focusing on the RCT data. Recently, the Society for Cardiovascular Angiography & Interventions (SCAI) expanded on these guidelines and shed some light into PFO closure in these clinical scenarios, including migraine, DCS, and desaturation syndromes.^3^ The discussion of these clinical syndromes that follows will attempt to characterize our understanding of these conditions and guide sensible management decisions.

## PFO and migraines

### Introduction

Migraine headaches (MH) are a common disorder that affects about 6% of men and 18% of women with a high disability rate and heavy disease burden.^4^ The connection between strokes in young patients and the presence of an intracardiac RLS was well characterized by the 1990s. Ischemic strokes were also found to have an association with MH and were considered a risk factor for strokes. In 1998, Del Sette et al studied the relationship between RLS and migraines using transcranial Doppler in 44 patients with migraines with aura, 73 young patients with ischemic strokes, and 50 controls. They found a statistically significant difference in the presence of RLS in these groups compared with control. Forty-one percent of patients with migraines with aura showed evidence of an RLS whereas only 16% of controls showed evidence of an RLS.^5^ Further evidence of this association was suggested in a retrospective study of 37 patients who underwent transcatheter interatrial shunt closure for nonmigraine-related reasons. Postprocedural interviews showed that 18/21 of the patients with a history of migraines reported either complete resolution of migraines (10) or improvement in migraines (8), with only 3 patients reporting no changes in symptoms.^6^ This prompted further investigation of this connection and the possibility of adding PFO closure as a treatment for refractory migraines.

### Pathophysiology

A major limitation of any study regarding MH is the heterogeneity in the causative mechanisms of migraines. The diagnosis of MH with or without aura describes a constellation of symptoms rather than a focal cause. MH may have multiple etiologic mechanisms making a single treatment approach difficult. One prominent theory is that migraines are due to triggering of the nociceptive receptors of the meninges and their blood vessels. This conducts down multiple interconnected neuronal pathways, the largest of which is the trigeminal neurovascular system resulting in MH with aura. One potential explanation of the association of MH with PFO is that an RLS allows for vasoactive substances (eg, serotonin, prostaglandins, and nitric oxide) to bypass degradation in the pulmonary capillaries and enter the cerebral circulation to trigger this nociceptive pathway. Other theories include the shunting of microemboli such as platelet aggregates, which disrupt cerebral circulation and autoregulation. Additionally, the presence of an RLS has also been thought to cause lower cerebral blood flow and trigger MH.^7^ More recently, it has been shown that patients with PFO and MH have higher levels of oxidative stress that triggers both serotonin and tissue factor pathways leading to higher levels of thrombin generation and higher risk of microemboli. Importantly, it has been shown that this upregulation is potentially reversible with PFO closure.^8^

### Evidence

There have been 3 RCTs to investigate the potential effects of PFO closure in patients with MH (Table 1). The Migraine Intervention With STARFlex Technology (MIST) trial (2007) was the first RCT of PFO closure for refractory MH. This double-blinded study compared transcatheter PFO closure with a STARFlex device (NMT Medical) to a sham procedure.^9^ The results are controversial with limited generalizability, and the STARFlex device is no longer manufactured.^10^

**Table 1:** Summary of major randomized control trials on PFO closure for migraines.

Trial (Reference, year)	Notable inclusion criteria	Treatment/control group (total patients)	Closure device(s) used	Control method	Notable outcomes	Comments
PRIMA (Mattle et al,^11^ 2016)	Chronic migraines sometimes with aura, failed at least 2 classes of medications	53/54 (107)	Amplatzer PFO Occluder	Aspirin/clopidogrel	No statistical differences in monthly migraine days (with and without aura). Closure patients were more often “responders”	Subgroup analysis: significantly fewer attacks and days of migraines specifically with aura
PREMIUM (Tobis et al,^12^ 2017)	Chronic migraines sometimes with aura, failed at least 3 classes of medications	123/107 (220)	Amplatzer PFO Occluder	Sham procedure (complete procedure including placement of guide wire in left atrium without PFO closure)	Closure group with more reduction in migraine days and complete cessation of attacks	Large number of “responders” in both groups, subgroup analysis: higher responder rate of migraines with aura
Pooled analysis (Mojadidi et al,^13^ 2021)	PRIMA (2016) and PREMIUM (2017) trials	176/161 (337)	Amplatzer PFO Occluder	See above	Reduction in monthly migraine days, attacks, and complete cessation	Subgroup analysis: statistical reduction was driven by a decrease in migraines with frequent aura

PFO, patent foramen ovale.

The PRIMA trial (2016) was an RCT that included 107 patients with chronic MH with aura who had failed at least 2 medications and were found to have a PFO. Patients were randomized to the closure (53 patients, Amplatzer PFO Occluder [St. Jude Medical]) or control (54 patients, no sham procedure) group. A “responder” was defined as a patient with >50% reduction in migraine days. The study showed no statistically significant change in the primary end point of monthly migraine days but did show that PFO closure patients were more often “responders” (38% vs 15%; *P* = .02). Further subgroup analysis of migraines with aura showed fewer migraine days (−2.4 vs −0.6; *P* = .01), fewer attacks (−2.0 vs −0.5; *P* = .01), and more complete resolution of migraines with aura (40% vs 10%; *P* = .004).^11^

The Premium Trial-Prospective, Randomized, Investigation to Evaluate Incidence of Headache Reduction in Subjects with Migraine and PFO comparing Using the AMPLATZER PFO Occluder to Medical Management (PREMIUM) trial (2017) was the largest and most rigorous RCT on this subject. It included 220 patients with MH who had failed at least 3 different medications and had either grade 4 or 5 RLS on transcranial Doppler (>100 bubbles/min). All patients were then pretreated with aspirin 325 mg and clopidogrel 600 mg, sedated, and underwent the initial steps of a closure procedure including femoral venous access and the probing of the interatrial septum (IAS) with a J-tipped guide wire. Only those patients in whom a PFO was proven by the presence of the guide wire in the left atrium and confirmed on intracardiac echo were then randomized to closure (123 patients, Amplatzer PFO Occluder) or control (107 patients, procedure concluded without intervention). The primary efficacy end point was the “responder rate” which was defined equivalently to the Percutaneous Closure of Patent Foramen Ovale in Migraine with Aura (PRIMA) trial as a patient with >50% reduction in migraine attacks. A large number of patients in both groups were characterized as “responders” (38.5% vs 32%; *P* = .32) without statistical differences between the groups. However, the PFO closure group had a significantly larger reduction in number of migraine days (−3.4 vs −2.0; *P* = .03) and complete cessation of migraine attacks (8.5% vs 1.0%; *P* = .01). Further subgroup analysis of migraines with frequent aura vs the control group showed a significantly higher responder rate (49% vs 23%; *P* = −.015) and complete cessation of attacks (15.4% vs 2.5%; *P* = .04).^12^

Importantly, although all RCTs did not meet statistical significance with respect to their primary efficacy end points, the secondary analysis of migraines with aura suggests a strong signal with PFO closure. A pooled analysis of patient level data from the PRIMA and PREMIUM trials revealed interesting observations. This larger dataset (337 total patients: 176 closure, 161 control) demonstrated decreased monthly migraine days (−3.1 vs −1.9; *P* = .02), decreased number of attacks (−2.0 vs −1.4; *P* = .01), and higher rates of complete migraine cessation (9% vs 0.7%; *P* < .001). Further subgroup comparisons between subgroups with frequent aura (>50% attacks with aura), infrequent aura, and without aura showed that this statistical reduction was driven by those with frequent aura receiving closure.^13^

The platelet aggregation pathway for MH was initially evaluated in a small cohort prospective analysis in 2005. In patients who experienced postprocedural migraines after PFO closure for nonmigraine-related reasons, the addition of clopidogrel to aspirin was effective in reducing migraine symptoms.^14^ A subsequent study by Sommer et al^15^ in 2018 analyzed data from 136 patients from a single physician’s office with migraines (with and without aura) and a PFO. Patients were treated with clopidogrel 75 mg daily without aspirin. A total of 80 patients had improvement of >50% reduction in migraines and were considered “responders” to clopidogrel. Of the remaining 56 patients, 45 consented to platelet reactivity testing and 19 were found to have inadequately inhibited platelets. These patients were switched to prasugrel with another 10 patients then classified as responders. All the responders were offered PFO closure. Fifty-six patients underwent PFO closure with either the GORE HELEX Septal Occluder (W.L. Gore & Associates) or GORE CARDIOFORM Septal Occluder (W.L. Gore & Associates). Of the PFO closure patients, 94% had persistent symptomatic improvement after 3 months when antiplatelet therapy was discontinued.^15^ A similar study using ticagrelor yielded similar positive results.^16^ The RELIEF trial is an RCT currently undergoing enrollment for P2Y12 inhibitor migraine responsiveness patients with a PFO and randomizing them to PFO closure using GORE Cardioform Septal Occluder or sham procedure.^17^

### Conclusion

PFO closure as a treatment for refractory MH remains a promising idea but is not ready for widespread adoption because of a lack of RCT data demonstrating its benefit. The FDA will generally not adjudicate the efficacy of a procedure based solely on a meta-analysis. Investigations into the effectiveness of PFO closure have largely yielded positive results in subgroup analyses, while primary outcomes in these few studies show no benefit. Studies are limited by the multifactorial causes of migraines and the heterogeneity of clinical presentations. Use of PFO closure as a treatment for migraines appears to be most promising in patients with frequent aura or those who are responsive to P2Y12 inhibitors. Ongoing clinical trials hope to identify particular patient subsets with MH who might benefit from PFO closure. Both the 2022 Guidelines from SCAI (conditional recommendation, moderate certainty of evidence) and the 2021 Guidelines from the European Society of Cardiology (ESC) (conditional recommendation) recommend against the routine use of PFO closure as a treatment for migraines but remark that it may be considered in compassionate use cases for refractory debilitating migraine treatment.^3^^,^^18^

## PFO closure for systemic conditions (platypnea-orthodeoxia syndrome and other conditions)

### Platypnea-orthodeoxia syndrome

#### Introduction

Platypnea-orthodeoxia syndrome (POS) is a rare condition in which patients are dyspneic and hypoxic in an upright position but experience relief with supine positioning. Described predominantly by case reports and case series, the condition is typically attributed to an intracardiac RLS. However, given the scarcity of data on this condition, there is no robust clinical trial data to support a standardized approach to diagnosis, prevention, or treatment. For more than 20 years, treatment has focused on management of intracardiac shunts. With the growth of transcatheter-based procedures, closure of these shunts has yielded promising results.

Furthermore, certain changes in a patient’s physiologic state throughout their life can precipitate a symptomatic RLS. Many patients warrant consideration of PFO closure despite the lack of randomized control data to support their specific diagnosis.

#### Pathophysiology

There are 2 main mechanisms theorized for POS. The first involves transient increases in venous return/pressures that may increase the interatrial pressure gradient and enhance an RLS. This, however, does not adequately explain the positional nature of the condition. The second mechanism suggests a positional anatomic distortion of the PFO that increases blood flow across the IAS in the upright position. The multiple subtypes of each anatomic subcategory have case reports to support their inclusion in this characterization. However, owing to the heterogeneity of the underlying mechanisms, there is no consensus on the causative mechanism for this condition.^19^

POS is sometimes noted during failed attempts to wean patients from mechanical ventilation. Coughing, sneezing, Valsalva maneuvers, and mechanical ventilation can alter venous return and right atrial pressures to cause an RLS across the IAS and lead to hypoxemia. Furthermore, respiratory failure and hypoxemia can alter pulmonary resistance and lead to increased right heart pressure. Diagnosis of this mechanism is often difficult due to the transient nature of the RLS but can sometimes be visualized on echocardiogram during these periods of time. However, pressure gradients by themselves do not adequately describe this condition.^19^^,^^20^ Interestingly, pulmonary pressures in most patients with POS are either normal or minimally elevated. Careful attention must be given to the location of the shunt in these patients as a similar aggregate of symptoms may be present in patients with only intrapulmonary shunts leading to the diagnosis of hepatopulmonary syndrome that would not benefit from atrial septal interventions.

Positional anatomic distortion of the IAS and surrounding anatomic structures offers an explanation for the positional differences in this condition. A 2014 study on the effects of varying fraction of inspired oxygen (FiO_2_) and patient positioning on PFO recruitment and shunting showed that at hypoxic levels of FiO_2_, supine positioning led to increased PFO recruitment and shunting. This was thought to be due to increased pulmonary pressures. However, at normoxic levels of FiO_2_, upright positioning led to the highest frequency of PFO recruitment. It was suspected that upright positioning stretched the atrial septum and positioned the inferior vena cava in such a way that venous return was preferentially directed toward the atrial septum. This increase in PFO recruitment was independent of right atrial pressures as well.^21^ Further subsets of anatomic variations that have previously been shown to contribute to POS include a prominent eustachian valve/Chiari network that diverts blood to the superior septum,^22^ dilated aortic root or ascending aortic aneurysm that alters the position of the IAS,^23^^,^^24^ vertebral changes including severe kyphosis,^25^ diaphragmatic paralysis,^26^ mediastinal shift from thoracic changes including after pneumonectomy,^27^ or severe tricuspid regurgitation with jet directed toward IAS.^28^^,^^29^ Further evidence suggests that anatomic and pressure differences may only explain a component of the shunting. The specific pattern of vortical blood flow in the right and left atria contributes to not only the likelihood of shunting but also the overall maintenance of PFO patency.^30^ POS is likely a heterogeneous disorder caused by any of the aforementioned abnormalities.^19^^,^^20^

#### Evidence

Clinical data on management of POS with shunt closure largely come from case series. These observational studies retrospectively describe the treatment course and outcomes of a small subset of patients over time (Table 2). Due to the infrequent nature of this disorder, it is unlikely that a prospective RCT could ever be accomplished.

**Table 2:** Summary of largest retrospective studies on PFO closure for platypnea-orthodeoxia syndrome (POS).

Reference, year	Data source	Total patients	Major causative mechanisms	Notable outcomes	Comments
Guérin et al,^31^ 2005	Nine French centers (1993-2001)	76	Pneumonectomy (46%), ascending aortic aneurysm (14%)	Complete resolution of hypoxia in 78% of patients	Only patients with a history of underlying lung pathology had persistent hypoxia
Mojadidi et al,^32^ 2015	UCLA (2001-2012)	17	Underlying lung pathology (59%), advanced treatment for pulmonary hypertension (24%)	65% of patients had either improvement or complete resolution of hypoxia	24% of patients had residual mild-moderate RLS after closure
Shah et al,^20^ 2016	Toronto General Hospital (1997-2015)	52	Pneumonectomy (19%), aortic dilation (15%), mixed pulmonary pathology (14%)	All patients had resolution of hypoxia	23% of patients were closed with a non-PFO closure device for various anatomic reasons
Castro et al,^22^ 2022	CHUPorto (Portugal) (2010-2020)	14	Atrial septal aneurysm (71%), aortic enlargement (57%)	All patients had improvement in dyspnea, and only 1 patient had persistent nocturnal hypoxia due to obstructive sleep apnea	Larger portion of patients with a history of stroke (43%)

PFO, patent foramen ovale; RLS, right-to-left shunt.

The largest case series of percutaneous closure of PFO with RLS for POS consisted of 78 patients from multiple different centers in a French registry from 1993 to 2001. The most common causative mechanisms were recent pneumonectomy (46%) and ascending aortic aneurysm (14%). All patients experienced documented hypoxemia with upright positioning (oxygen saturation [SpO_2_] dropping from 84.5% (supine) to >79.9% [upright]). Seventy-six patients underwent PFO closure (5 different devices used), and a total of 65 patients completed follow-up. After closure, baseline supine SpO_2_ increased to 95.1% with upright cyanosis now only present in 24% of patients, all of whom had underlying lung pathology. Furthermore, dyspnea severity grade decreased significantly after closure (2.7 to >1.0).^31^

A similar retrospective single-center study of patients treated from 1997 to 2015 found 52 patients in whom PFO closure was performed for POS. Prior to closure, average supine SpO_2_ was 93% and worsened to 81% with upright positioning despite 19 patients being on oxygen at the time of measurements. Pneumonectomy was the most common causative finding (19.2%), followed by aortic dilation (15.4%), and mixed pulmonary pathologies (13.5%). Of the patients, 38.5% had no specific causative mechanism identified. TEE confirmed an RLS by either color or agitated saline injection in all patients. Of the patients, 65.9% had an atrial septal aneurysm and about one-quarter of patients were noted to have prominent eustachian valve/Chiari network. Preprocedural right heart catheterization showed normal right heart and pulmonary pressures in all patients, and closure was performed with 5 different devices. Immediately after closure, upright SpO_2_ improved to 95.1% on room air on average for all patients. On follow-up, all patients remained asymptomatic without postural changes affecting SpO_2_.^20^

Two smaller case series have also been published. The first reported 14 patients at a single Portuguese center diagnosed with POS and a PFO between 2010 and 2020. Unlike the prior studies, many of the patients had concurrent strokes (42.8%). All patients had RLS noted on TTE. Aortic root enlargement/ascending aortic aneurysm was found in 57.1% of patients, and an atrial septal aneurysm was present in 71% of patients. Three different closure devices were used. All patients experienced improvement in dyspnea with upright SpO_2_ improving from 91.5% to >95.1% and resolution of need for supplemental oxygen in all but 1 patient.^19^

Mojadidi et al^32^ reported 17 patients between 2001 and 2012 diagnosed with POS and a PFO with RLS in whom follow-up was available. Unlike other studies, 58.8% of patients had significant underlying lung pathologies including chronic obstructive pulmonary disease and pulmonary hypertension with 4 patients on bosentan or sildenafil, and 23.5% of patients had diaphragmatic paralysis. Of note, after PFO closure, 23.5% had mild-moderate residual RLS on imaging. After closure, 64.7% of patients had either resolution of symptoms (34.5%) or an improvement in symptoms and oxygen requirements (29.4%). Surprisingly, there were no differences in pulmonary function testing between the responders and nonresponders. However, of the 4 patients on either bosentan or sildenafil for pulmonary hypertension, only 1 experienced complete resolution of symptoms while the remaining 3 had no change in symptoms.^32^ Overall, this study had a population with significantly more pulmonary comorbidities and did not have as many causative anatomic findings for POS.

#### Conclusion

POS is rare, and management is unclear given the heterogeneity of causative mechanisms and the lack of specific diagnostic criteria. However, in patients with POS, a PFO with RLS is commonly found. Other frequently found possibly causative anatomic findings include pneumonectomy/mediastinal shift, aortic root/ascending aortic enlargement, or atrial septal aneurysm. Although the data are limited, in the absence of significant pulmonary pathologies and the presence of normal pulmonary pressures, percutaneous transcatheter PFO closure appears to be an effective treatment method. The 2021 ESC guidelines (strong recommendation, level of evidence: C) and 2022 SCAI guidelines (conditional recommendation, very low certainty of evidence) support PFO closure in this patient population.^3^^,^^18^ In patients with concurrent pulmonary pathologies such as chronic obstructive pulmonary disease or pulmonary hypertension, the data are even more limited, and no clear recommendation can be made. Focus should be placed on atrial septal anatomy in these patients given the high prevalence of anatomic variants such as atrial septal aneurysms which can complicate transcatheter closure.^33^

### Hypoxemia due to RLS

Patients with a PFO and arterial desaturation syndromes may not meet all the criteria for POS but, nonetheless, have a mechanistically similar condition that causes intermittent shunting of deoxygenated blood across the IAS. Although large trials do not exist to support PFO closure in these cases, there have been numerous instances in which it has been performed successfully including but not limited to the following:

1. Carcinoid heart disease causing eccentric severe tricuspid regurgitation has been seen to cause significant RLS and hypoxemia that can be treated with PFO closure.^34^^,^^35^
2. Traumatic tricuspid valve chordal rupture causing severe regurgitation and shunt across a PFO was treated with PFO closure with resolution of the cyanosis.^36^
3. Exertional hypoxemia with a large Chiari network and large RLS has been successfully treated with PFO closure.^37^
4. Refractory hypoxemia and cardiogenic shock after an inferior ST elevation myocardial infarction with an right ventricular infarct and large PFO had significant improvement with PFO closure.^38^

The 2021 ESC guidelines give a conditional recommendation (Level of evidence: C) for PFO closure when it has been shown to “unequivocally and critically contribute to the arterial desaturation and symptoms.”^18^

### Cryptogenic nonstroke systemic circulation thromboembolism

The etiology of systemic circulation thromboembolism conditions including limb ischemia, organ infarct, and type 2 myocardial infarction is often unclear. In the presence of a PFO with the absence of atrial fibrillation, a left ventricular thrombus, or another clear etiology, the condition is often attributed to an RLS through the PFO. There have been no RCTs and no large observational trials regarding management of a PFO in these conditions. The 2019 ESC guidelines combined the recommendations for stroke with the recommendations for other systemic thromboemboli due to similar pathophysiologic mechanisms.^39^ However, they predominantly rely on data from stroke management including use of the Risk of Paradoxical Embolism score (RoPE score) which has only been validated with stroke as the associated condition.^40^ The 2022 SCAI recommendations more specifically address systemic thromboembolism without a history of PFO-associated stroke by giving a conditional recommendation supporting PFO closure (very low certainty of evidence).^3^

There have been some suggestions that obstructive sleep apnea has a higher incidence rate of PFO and that episodes may lead to transient hemodynamic shifts that can facilitate RLS, hypoxemic episodes, and left circulation thromboembolic disease including strokes. Although, large studies are lacking in this area, PFO closure has been used in this area to mixed results.^41^, ^42^, ^43^, ^44^, ^45^, ^46^, ^47^

## PFO closure for DCS

### Introduction

Decompression illness describes the multiple different detrimental effects due to rapid depressurization most commonly during Self-Contained Underwater Breathing Apparatus diving (SCUBA). It can be split into 2 main entities, DCS and arterial gas embolism (AGE). DCS involves the release of gas dissolved into blood, joints, and various tissues creating tiny bubbles leading to tissue-specific injuries. AGE describes the process in which these released gas bubbles become venous gas emboli (VGE) and are shunted by various processes to the arterial circulation to cause a variety of deleterious effects. DCI was first described in miners and tunnel workers as “the bends” but has since been more commonly associated with divers, high-altitude aviators, and astronauts.^18^^,^^48^ With the rapid growth of SCUBA diving, there has been a search for methods for the primary and secondary prevention of DCI. A variety of denitrogenation, depressurization, and repressurization protocols have been established to help mitigate this risk.^49^ Given the pathophysiologic implications of an AGE, closure of PFO has been a focus of much of this investigation and treatment, with the prevalence of a PFO in divers with a history of DCI being reported as high as 97%.^50^

### Pathophysiology

Humans have evolved to function at normal atmospheric pressure (1 atm, 760 mm Hg). However, in modern times, hyperbaric and relatively hyperbaric conditions are widely prevalent including underwater recreational and construction activities, tunnel/mining activities especially when fed by an air compressor, high-altitude aircraft, or during extravehicular maneuvers in space flight. DCS is common in these fields, with 75% of military high-altitude U-2 pilots experiencing DCS at some point during their careers.^48^

The law of partial pressures describes that when transitioning to a relatively low-pressure environment, a portion of dissolved gases will exit the solution and turn into microscopic or macroscopic bubbles. These bubbles can then cause local trauma including joint disruption, pulmonary barotrauma, nerve/central nervous system damage, and skin damage (cutis marmorata). This condition can even progress to shock, circulatory collapse, or death. There are 3 categorizations of DCS. Type 1 involves pain in the muscles and joints with localized paresthesia. Type 2 involves systemic effects including central nervous system impairment (impaired consciousness, vestibular symptoms, paralysis), cardiopulmonary effects, shock, and death. The third type is cutis marmorata which is a bruise-like reticular pattern on the skin that can be extremely painful and may indicate severe underlying damage. Typically, VGE are filtered by the lungs, and in the absence of a large burden of venous gas, the body compensates without significant ill effects. However, in patients with RLS, the systemic effects can be more onerous, especially considering the high prevalence of PFO in the general population (∼25% of the general population).^18^^,^^51^^,^^52^ NASA has developed methods of prevention of DCI including protocoled denitrogenation of astronauts prior to extravehicular activities and operating their extravehicular suits at specific hypobaric pressures to limit the pressure differential. The goal of this process is to eliminate VGE, which has a 98% negative predictive value for the subsequent development of DCS.^51^^,^^52^ As an alternative to these denitrogenation protocols, which are not broadly available, percutaneous PFO closure has been proposed as a way of lowering the risk of DCI, especially in secondary prevention.

There are 2 important points to note regarding DCS. First, DCI is largely a clinical diagnosis defined by a history of recent high-risk activity and multiple clinical factors. There are no specific, widely accepted diagnostic criteria for this disorder. This fact complicates any study focused on treatment modalities for DCI. Furthermore, PFO closure is theorized to specifically prevent AGE that are most commonly responsible for type 2 systemic effects. As a result, PFO closure may not prevent all types of DCI.

### Evidence

Studies have previously shown that simulated depressurization events lead to the appearance of arterial gas.^53^ For decades, physicians have been performing transcatheter closure of PFO as a treatment for secondary prevention of DCI given the intuitive logic of this treatment in such patients. However, published data on PFO closure for DCI largely consists of small case series, most of which lack an adequate control group.^49^ A meta-analysis of small studies demonstrated that there is an odds ratio of 5.63 for DCS in the presence of an RLS.^18^ However, prospective RCT data are lacking (Table 3).

**Table 3:** Summary of limited studies on PFO closure for decompression illness and sickness. (DCI/DCS).

Reference, year	Data source	Total patients	Subgroups	Notable outcomes	Comments
Honěk et al,^54^ 2014	Single center, 183 consecutive divers (2006-2013)	47 with grade 3 PFO	PFO closed, history of DCS: 20 Not closed, history of DCS: 13 Not closed, no history of DCS: 14	Two dive protocols followed (A/B) (Closure dive A/B vs Control dive A/B) Similar rates of VGE: 80%/100% vs 74%/88%, *P* = 1.0/1.0 Lower rates of AGE: 0%/0% vs 32%/88%, *P* = .02/<.01	The study showed that PFO closure prevents AGE, but did not examine the subsequent development of DCS rates
DIVE-PFO,^56^ 2022	Single center, 829 consecutive divers (2006-2018)	702 available for analysis	PFO closed, grade 3 PFO: 55 Not closed, conservative diving: -grade 3 PFO: 98 -grade 1/2 PFO: 128 Unrestricted dive profile, No PFO controls: 421	Rates of DCS per 1000 dives (Before vs >After intervention) (Mean follow-up 6.5 y) Conservative dive profile: -grade 3 PFO: 5.23 vs >0.75, *P* < .0001 -grade 1/2 PFO: 1.76 vs >0.28, *P* = .0005 PFO Closure with grade 3 PFO: 4.13 vs >0.00, P < .0001	The presence of PFO was found to be a major risk factor for DCS Conservative dive profiles effectively lower rates of DCS PFO closure may be able to drastically lower the risk of DCS

AGE, arterial gas emboli; PFO, patent foramen ovale; VGE, venous gas emboli.

A small 2014 study looked at the presence of venous and arterial gas emboli in 47 grade 3 PFO patients (shunted bubbles which were too numerous to count) undergoing simulated dives in a hyperbaric chamber. Twenty patients with a history of DCS underwent PFO closure with either Amplatzer Septal Occluder or Occlutech Figulla PFO Occluder N (Occlutech International AB). Twenty-seven grade 3 PFO patients were used as controls of which 13 had a history of DCS. They followed 2 different diving protocols and measured the presence of VGE and AGE using TTE and transcranial doppler. Venous bubbles were widely present at similar rates between the closure group and the control group in the 2 dives (80%/100% vs 74%/88%; *P* = 1.0/1.0) indicating predictable production of VGE with dives. However, arterial bubbles were completely absent in the closure group compared with the control group in both dives (0%/0% vs 32%/88%; *P* = .02/<.01). Fewer symptoms of DCS were present in the closure group, although this difference did not reach significance (0% vs 25%; *P* = .49).^54^ This study provided direct evidence that not only does decompression cause venous and arterial gas emboli, but that AGE are preventable with PFO closure. However, it did not provide direct evidence that closure prevents DCS.

The DIVE-PFO registry is the largest prospective nonrandomized study of PFO for the prevention of DCI. The registry screened 829 divers for the presence of an RLS. One hundred fifty-three patients were found to have a grade 3 PFO and considered a high-grade shunt whereas 128 patients were found to have a grade 1 or 2 shunt (low-grade). Patients with a high-grade shunt were offered PFO closure of which 55 underwent closure (either Amplatzer Septal Occluder or Occlutech Figulla PFO Occluder N). The remaining patients (98 with high-grade RLS and 128 with low-grade RLS) were managed with a conservative diving profile that limits ascent rates, maximum depth, dive time, and number of dives per day. Conservative diving profiles have been previously shown to lower VGE formation.^54^^,^^55^ Mean follow-up was 6.5 years. Groups were compared with 421 control patients without an RLS using an unrestricted dive profile. The results showed that a conservative diving profile effectively lowered but did not eliminate the incidence rate (per 1000 dives) of DCS in low-grade (1.76 vs >0.28, *P* = .0005) and high-grade (5.23 vs >0.75, *P* <.0001) RLS patients. No DCS events occurred after PFO closure (4.13 vs >0.00, *P* < .0001).^56^ This is the largest study to date that provides evidence that a conservative diving profile can lower the incidence rate of DCS and PFO closure may be able to eliminate that risk.

### Conclusion

DCI and arterial gas emboli are characterized by both local and systemic effects. Type 2 DCS can lead to profound cardiopulmonary collapse and even death. Despite the rarity of circumstances that would expose a typical patient to a risk of decompression, certain high-risk occupations and recreational activities can carry a high lifetime risk of DCI. As in most disease processes, prevention is paramount including activity avoidance, the use of conservative dive profiles, prebreathing high concentrations of oxygen, and denitrogenation. Some studies also suggest that lifestyle and behavioral changes such as smoking/alcohol cessation, weight loss, and adequate hydration can also mitigate risk.^18^ There is not enough data to recommend primary screening for PFO in all patients undergoing high-risk activities such as diving or high-altitude flights. However, the limited data does suggest that PFO closure may be a reasonable option for secondary prevention of DCI in patients who wish to continue high-risk activities. The 2022 SCAI guidelines give a conditional recommendation (very low certainty of evidence) against routine closure for DCI due to the lack of randomized data and the potential benefits of nonclosure-related changes as discussed above.^3^ The 2021 ESC guidelines however do note that in patients who cannot realistically perform or are unwilling to perform low-risk dive maneuvers, PFO closure may be proposed, and unrestricted diving should not be resumed until the PFO has been sealed (strength of statement: strong, level of evidence: C).^18^

## PFO closure prior to liver transplantation

### Introduction

Cardiovascular complications are the second highest cause of nongraft-related death in the first year after liver transplantation.^57^ Given the high prevalence of a PFO in the general population, a portion of patients undergoing liver transplantation evaluation for end-stage liver disease (ESLD) will be incidentally noted to have a PFO. During liver transplantation, there are hemodynamic shifts that theoretically increase the likelihood of a transient RLS in the presence of a PFO. This in turn can conceptually increase the risk of a perioperative stroke. Large studies on the subject have not been performed, but case reports have demonstrated the possibility of these paradoxical emboli.^58^^,^^59^ Only retrospective analyses have investigated the risks associated with PFO prior to liver transplantation. As a result, when a PFO is discovered in this patient population, the question is raised of pretransplant preventive closure to lower stroke risk.

### Pathophysiology

End-stage liver disease patients have increased levels of peripheral nitric oxide. This leads to both splanchnic and peripheral vasodilation which causes relative splanchnic hypervolemia and central hypovolemia, which is the driving force behind the decreased systemic vascular resistance and a hyperdynamic circulatory state in ESLD patients.^60^ This hyperdynamic state causes increased pulmonary and systemic blood flow. Liver transplantation requires clamping of the hepatic vein. This process causes a significant drop in cardiac preload. However, once the transplant has been secured and the hepatic vein is allowed reperfusion, there subsequently is a marked increase in venous return. In patients with a PFO, this drastic transition to a hypervolemic right heart state is enough to cause an RLS, although often transient.^61^

Furthermore, during transplantation, there is a risk of air, debris, and clot formation in this venous system which can potentially travel to the heart. The sudden reperfusion of the right heart leads to a risk of pulmonary embolism or, in the case of an RLS, systemic embolism and stroke. Ellis et al published a case series of 16 patients who underwent TEE at the time of liver transplantation and found evidence of transient right heart congestion, 2 patients with paradoxical embolism, and 1 patient with a right atrial thrombus.^61^

### Evidence

There have been 3 relatively small retrospective analyses of outcomes in liver transplant patients with a PFO (Table 4). Alba et al retrospectively studied 736 liver transplant patients between 2000 and 2008 and found 27 to have a PFO (3.7%). When compared with a control cohort from the same period, there were no significant differences in outcomes with respect to stroke, 30-day mortality, days of mechanical ventilation, dialysis, delirium, or Acute physiology and Chronic Health Enquiry score II (APACHE II) scores. The only stroke in a PFO patient was a hemorrhagic nonembolic stroke. No pulmonary emboli were noted in the study population. A significant criticism of this study was the low prevalence of PFO in this patient population compared with the general population.^62^

**Table 4:** Summary of largest retrospective studies on outcomes of liver transplantation with a PFO. All trials used a control cohort for comparison.

Reference, year	Data source	PFO patients/Total patients	PFO prevalence in the cohort	Notable outcomes	Comments
Alba et al,^62^ 2011	Toronto General Hospital (2000-2008)	27/736	3.7%	No significant differences in stroke, 30-day mortality, days of mechanical ventilation, dialysis, delirium, or APACHE II scores	Lower than expected PFO prevalence, only 1 stroke occurred (hemorrhagic/nonembolic)
Werlang et al,^63^ 2016	Mayo Clinic (Florida) (2008-2013)	97/935	10.4%	No significant differences in stroke, length of ICU stay, days of mechanical ventilation, postoperative oxygen requirements, and 30-day mortality	Surgeons used “piggyback” technique to lower the risks of embolism
Gertsvolf et al,^64^ 2018	USC (2010-2016)	46/350	13.1%	No significant differences in stroke, major adverse cardiovascular events, death, myocardial infarction, congestive heart failure, or arrhythmia	Only 1 stroke occurred in a PFO patient (hemorrhagic/nonembolic). 2/5 strokes in non-PFO patients were likely due to embolism

PFO, patent foramen ovale.

Werlang et al performed a similar retrospective analysis of 935 liver transplant patients between 2008 and 2013 and found 97 to have a PFO (10.4%). The prevalence of PFO in this study was higher than in the Alba et al study but remained lower than in the general population. When compared with a matched control group from the same period, both groups had similar rates of stroke, length of ICU stay, days of mechanical ventilation, postoperative oxygen requirements, and 30-day mortality. Of note, the surgeons in their program used a “piggyback” technique for their liver transplantations which preserved the native retrohepatic vena cava and theoretically lowered the risks of embolism.^63^

Gertsvolf et al published the most recent study of 350 liver transplant patients between 2010 and 2016 of which 46 had a PFO (13.1%). When compared with a non-PFO control cohort, there were no significant differences in outcomes. A total of 6 in-hospital strokes occurred, 1 in a PFO patient and 5 in non-PFO patients. The stroke in the PFO patient was a hemorrhagic nonembolic stroke and 2/5 strokes in non-PFO patients were likely due to embolism. Mortality rates and other long-term clinical events were similar between the 2 groups.^64^

### Conclusion

The reperfusion phase of liver transplantation theoretically leads to a risk of paradoxical embolism in patients with a PFO, given the rapid change in right heart pressures and the likelihood of air and other debris entering the right heart. Although case reports have shown this possibility, the 3 largest retrospective studies on liver transplantation patients with PFOs do not show any significant differences in outcomes especially strokes when compared with control patients without PFOs. These studies, however, only comprise a combined 170 PFO patients with only 2 strokes noted in PFO patients, both of which were hemorrhagic and nonembolic.^62^, ^63^, ^64^

Percutaneous PFO closure carries risks that are quite small in the general population but are elevated in patients with coagulopathic ESLD.^1^ Some authors suggest an algorithmic approach to PFO closure prior to liver transplantation.^59^^,^^63^ However, the literature does not support routine closure of asymptomatic PFO prior to liver transplantation. It is reasonable though to consider PFO closure in a patient prior to liver transplantation if they have other indications for PFO closure such as history of paradoxical embolism, cryptogenic stroke, or POS. These findings are consistent with the available data on the risks associated with an incidentally discovered PFO during and after lung transplantation that have not shown clear benefit of intervention prior to or during surgery.^65^

## Other considerations

The FDA has given approval to multiple devices for PFO closure in the setting of cryptogenic stroke from presumed paradoxical embolism.^66^^,^^67^ However, the expansion of PFO closure to nonstroke indications has not yet received FDA approval, and the pursuit of closure in these circumstances should be considered off-label (Central Illustration). Care must be taken to ensure the patient has an adequate understanding of the risks, benefits, and complications associated with PFO closure for these indications in addition to the occasional difficulties associated with health insurance coverage of the procedure.

**Central Illustration:**
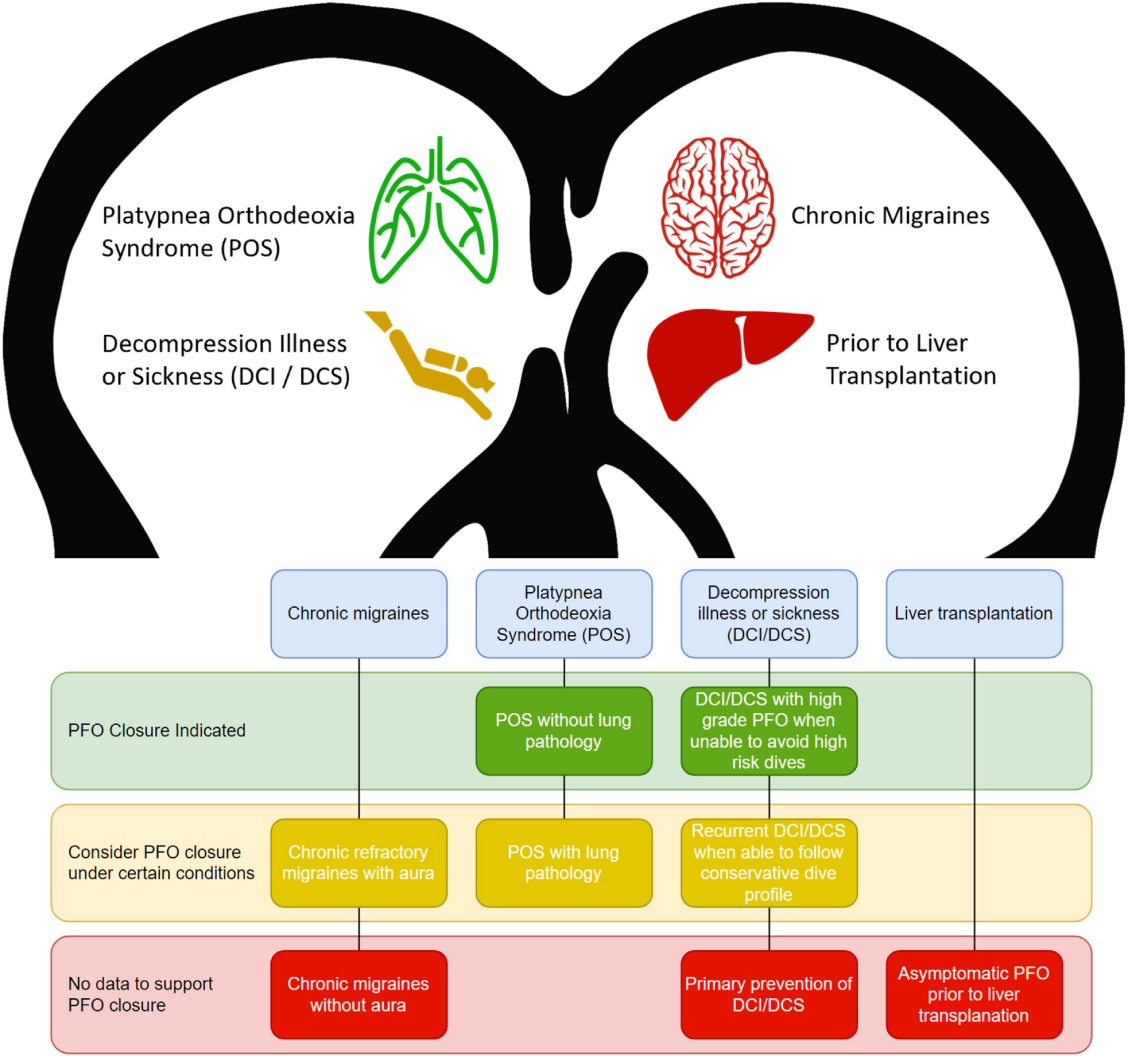
**A summary of recommendations for patent foramen ovale (PFO) closure for nonstroke indications.** PFO closure indicated (green); consider PFO closure under certain conditions (yellow); no data to support PFO closure (red).
